# The prognostic value of C-X-C motif chemokine receptor 4 in patients with sporadic malignant peripheral nerve sheath tumors

**DOI:** 10.1186/s40880-017-0246-z

**Published:** 2017-10-11

**Authors:** Chao Zhang, Fang-Yuan Chang, Wen-Ya Zhou, Ji-Long Yang

**Affiliations:** 1 Department of Bone and Soft Tissue Tumor, Tianjin Medical University Cancer Institute & Hospital, Tianjin, 30060 P. R. China; 2 National Clinical Research Center for Cancer, Tianjin Medical University Cancer Institute & Hospital, Tianjin, 300060 P. R. China; 3 Key Laboratory of Cancer Prevention and Therapy, Tianjin Medical University Cancer Institute & Hospital, Tianjin, 300060 P. R. China

**Keywords:** Sporadic malignant peripheral nerve sheath tumor, C-X-C motif chemokine receptor 4 (CXCR4), C-X-C motif chemokine ligand 12 (CXCL12), Cyclin D1

## Abstract

**Background:**

Recent studies indicate that C-X-C motif chemokine receptor 4 (CXCR4) and its ligand, C-X-C motif chemokine ligand 12 (CXCL12), stimulate expression of the cell cycle regulatory protein Cyclin D1 in neurofibromatosis 1-associated malignant peripheral nerve sheath tumor (MPNST) cells and promote their proliferation. In this study, we measured the expression of CXCR4, CXCL12, and Cyclin D1 proteins in sporadic MPNST tissues from Chinese patients and investigated their prognostic values.

**Methods:**

CXCR4, CXCL12, and Cyclin D1 protein expression in samples from 58 Chinese patients with sporadic MPNST was assessed with immunohistochemical staining. Their prognostic values were evaluated with Kaplan–Meier analysis and a log-rank test. Multivariate Cox regression analysis was used to identify independent prognostic factors.

**Results:**

High expression of CXCR4, CXCL12, and Cyclin D1 was observed in 19 (32.8%), 32 (55.2%), and 16 (27.6%) samples, respectively. CXCR4 expression was positively correlated with CXCL12 expression (*r* = 0.334, *P* = 0.010) and Cyclin D1 expression (*r* = 0.309, *P* = 0.018). Patients with high CXCR4 expression showed longer overall survival than those with low CXCR4 expression (χ^2^ = 4.642, *P* = 0.031).

**Conclusion:**

High CXCR4 expression may define a specific subtype of sporadic MPNST with favorable prognosis.

## Background

Malignant peripheral nerve sheath tumors (MPNSTs) are highly aggressive cancers that account for approximately 5%–10% of all soft tissue sarcomas [1]. MPNSTs are defined as tumors originating from or differentiating into nerve sheath cells [2], with the exception of tumors originating from the epineurium or peripheral nerve vasculature [3]. MPNSTs may arise sporadically (40%), following radiotherapy (10%), or in association with neurofibromatosis type 1 (NF1) (40%–50%) [4, 5]. NF1 is caused by the mutation of *NF1* gene. The *NF1* gene (located in 17q11.2) product is neurofibromin. A region of neurofibromin functions as a GTPase-activating protein shown to be involved in negative regulation of the RAS pathway, then caused the hydrolyzation and inactivation of GTP [6]. The mutations of *NF1* cause neurofibromin to lose its functions as a tumor-suppressing factor. Types of *NF1* mutations include frameshift, nonsense, missense, splicing alteration and deletion, and loss of heterozygosity [7]. The MPNST that develops from benign NF1 is named NF1-associated MPNST and is the main cause of death among NF1 patients [7, 8]. Compared with sporadic MPNSTs, NF1-associated MPNSTs usually develop in young adults, frequently relapse (in approximately 50% of patients), and lead to a dismal prognosis partly because of the high mutation frequency of *NF1* gene [8]. Traditional treatment modalities, such as surgery, chemotherapy, and radiotherapy, have limited therapeutic effects on MPNSTs [9, 10]. Sporadic MPNSTs have some unusual characteristics. For instance, high expression of fibroblast growth factor receptor 1 (FGFR1) is an independent prognostic predictor for long overall survival (OS) in patients with sporadic MPNST, but associated with poor prognosis of several other types of epithelial cancer [11].

Chemokines are a class of small molecules that bind to G protein-coupled receptors on target cells. Chemokine-induced signaling cascades regulate leukocyte migration, hematopoietic stem cell proliferation, and cell adhesion to extracellular matrix molecules [12, 13]. C-X-C motif chemokine ligand 12 (CXCL12), also known as stromal cell-derived factor-1, is a chemokine that binds to C-X-C motif chemokine receptor 4 (CXCR4). CXCR4/CXCL12 signaling plays important roles in the maintenance of embryonic development, immune and inflammatory responses, regulation of hematopoiesis, human immunodeficiency virus infection, and induction of angiogenesis [14, 15]. Signaling triggered by the CXCR4-CXCL12 binding and subsequent regulation of Cyclin D1 expression has been shown to regulate several biological functions in cancers, such as cell proliferation, tumor invasion and metastasis, angiogenesis, and cell–microenvironment interactions [16, 17]. It is reported that CXCR4 and CXCL12 mRNA expression was reduced in hepatocellular carcinoma and renal cancer compared with non-cancerous tissues, but unchanged in most digestive tract carcinomas, such as colon esophageal or gastric cancer [18]. At the same time, high levels of CXCR4 have been associated with short OS in breast cancer patients [19, 20]. According to the divergence of CXCR4 expression in different cancers, extensive analyses of CXCR4 expression levels and functional studies will be necessary to understand the precise roles of CXCR4/CXCL12 signaling in different cancer types.

In NF1-associated MPNST, CXCR4/CXCL12 signaling stimulates Cyclin D1 expression and promotes cell cycle progression by activating the AKT/serine–threonine kinase/glycogen synthase kinase 3 beta signaling pathway, ultimately increasing MPNST cell proliferation. Suppression of CXCR4 activity decreases MPNST cell growth and inhibits tumorigenesis in allografts and in spontaneous genetic mouse models of MPNST [16]. However, the role of CXCR4 in sporadic MPNSTs is unclear. In this study, we addressed this question by measuring the expression of CXCR4, CXCL12, and Cyclin D1 proteins in tumor samples from Chinese patients with sporadic MPNST.

## Methods

### Patients and tissue samples

The clinical records of MPNST patients diagnosed between January 6th, 1991 and December 1st, 2011 were evaluated to include only patients with sporadic MPNST and to exclude patients with NF1-associated or radiotherapy-induced MPNST. Clinicopathologic information was collected, including sex, age, tumor site, tumor size, American Joint Committee on Cancer (AJCC) stage, radiotherapy, chemotherapy, surgery type, disease recurrence, metastasis, and patient survival.

Tissue samples were obtained from the Department of Pathology, Tianjin Medical University Cancer Institute and Hospital. All tissues were collected before patients received radiotherapy and/or chemotherapy and were formalin-fixed and then embedded in paraffin. Two independent pathologists confirmed the diagnosis and ensured that at least 90% of cells in each specimen were tumor cells.

### Tissue microarrays and immunohistochemistry

Tissue microarrays (TMAs) were constructed with the most representative areas of the tumor region as previously reported [21, 22]. Immunohistochemical (IHC) staining of TMAs was performed using the streptavidin–peroxidase method [23] with the following biotinylated primary antibodies: anti-CXCR4 antibody (1:100, ab124824, Abcam, Cambridge, UK), anti-CXCL12 antibody (1:100, sc-28876, Santa Cruz Biotechnology, Santa Cruz, CA, USA), and anti-Cyclin D1 antibody (1:50, Beijing Zhong Shan Golden Bridge Biotechnology Co. Ltd, Beijing, China).

The stained specimens were evaluated independently by two pathologists who were blinded to the clinicopathologic information and patient prognoses. Slides were examined by light microscopy, and high-power fields of five random areas per tumor were evaluated. Staining intensity was scored as 0 for no staining, 1 for light yellow, 2 for brownish yellow, and 3 for brownish black staining. The percentage of positive cells was scored as 0 for ≤ 10%, 1 for 11%–25%, 2 for 26%–50%, 3 for 51%–75%, and 4 for > 75%. The summation of the two scores was taken as the final IHC score: 0–1 (negative), 2–3 (weak positive), 4–5 (moderate positive), and 6–7 (strong positive). The expression level was further categorized into low (negative and weak positive) and high (moderate and strong positive) expression based on the final IHC score.

### Statistical analysis

Associations between CXCR4, CXCL12, and Cyclin D1 expression levels and clinicopathologic variables were evaluated using the χ^2^ or Fisher’s exact tests. Correlations among the three protein levels were determined using Pearson’s correlation coefficient. The follow-up was stopped at April 14th, 2015. OS was defined as the duration from the date of diagnosis to the date of death or the last follow-up. Disease-free survival (DFS) was defined as the duration from the date of diagnosis to the confirmed disease progression or the last follow-up. Relationships between CXCR4, CXCL12, or Cyclin D1 expression levels and survival were evaluated using the Kaplan–Meier method and a log-rank test. The data of patients who were lost to follow-up or dead because of non-tumor-related diseases were censored. The Cox regression model was used for univariate and multivariate analyses. Analyses were performed using SPSS 19.0 software for Windows (SPSS Inc., Chicago, IL, USA). A *P* value < 0.05 was considered statistically significant.

## Results

### Clinicopathologic characteristics of sporadic MPNST patients

A total of 58 patients with sporadic MPNST, 31 males and 27 females with an average age of 46.5 years (median, 46 years; range 6–86 years), were selected (Table 1). At the end of follow-up, 22 patients showed no evidence of tumor recurrence or metastasis, whereas 36 had disease progression as determined with imaging or pathologic evidence of recurrence or metastasis. Thirty-one patients died of MPNST. The median DFS was 29 months (range 0–149 months). The 5- and 10-year DFS rates were 32% and 28%. The median OS was 109 months (range 0–150 months). The 5- and 10-year OS rates were 59% and 29%.

**Table 1:** Relationships between CXCR4, CXCL12, and Cyclin D1 expression levels and clinicopathologic characteristics of sporadic MPNST patients

Clinical characteristic	Total (cases)	CXCR4 expression [cases (%)]				CXCL12 expression [cases (%)]				Cyclin D1 expression [cases (%)]			
		High	Low	χ^2^	*P*	High	Low	χ^2^	*P*	High	Low	χ^2^	*P*
Sex				0.420	0.517			1.008	0.315			2.259	0.133
Male	31	9 (29.0)	22 (71.0)			19 (61.3)	12 (39.7)			6 (19.4)	25 (80.6)		
Female	27	10 (37.0)	17 (63.0)			13 (48.1)	14 (51.9)			10 (37.0)	17 (63.0)		
Age (years)				0.105	0.745			1.277	0.258			0.840	0.359
≥ 40	38	13 (34.2)	25 (65.8)			23 (60.5)	15 (39.5)			9 (23.7)	29 (76.3)		
< 40	20	6 (30.0)	14 (70.0)			9 (45.0)	11 (55.0)			7 (35.0)	13 (65.0)		
Tumor site				2.105	0.349			0.512	0.774			1.045	0.593
Head/neck	10	3 (30.0)	7 (70.0)			5 (50.0)	5 (50.0)			2 (20.0)	8 (80.0)		
Trunk	23	10 (43.5)	13 (56.5)			14 (60.9)	9 (39.1)			8 (37.8)	15 (62.2)		
Extremity	25	6 (24.0)	19 (76.0)			13 (52.0)	12 (48.0)			6 (24.0)	19 (76.0)		
Tumor size (cm)^a^				< 0.001	1.000			1.612	0.447			0.397	0.820
< 5	21	7 (30.0)	14 (70.0)			14 (70.0)	7 (30.0)			5 (23.8)	16 (76.2)		
5–10	21	7 (30.0)	14 (70.0)			11 (52.4)	10 (47.6)			6 (28.6)	15 (71.4)		
> 10	15	5 (33.3)	10 (66.7)			7 (46.7)	8 (53.3)			5 (33.3)	10 (66.7)		
AJCC stage^b^				2.400	0.494			1.679	0.642			2.484	0.478
I	6	1 (16.7)	5 (83.3)			3 (50.0)	3 (50.0)			2 (33.3)	4 (66.7)		
II	32	13 (40.6)	19 (59.4)			20 (62.5)	12 (37.5)			9 (28.1)	23 (71.9)		
III	7	2 (28.6)	5 (71.4)			4 (57.1)	3 (42.9)			3 (42.9)	4 (57.1)		
IV	10	2 (20.0)	8 (80.0)			4 (40.0)	6 (60.0)			1 (10.0)	9 (90.0)		
Radiotherapy^c^				0.196	0.658			0.252	0.615			0.163	0.686
Yes	21	6 (28.6)	15 (71.4)			11 (52.4)	10 (47.6)			7 (33.3)	14 (66.7)		
No	32	11 (34.3)	21 (65.7)			19 (59.4)	13 (40.6)			9 (28.1)	23 (71.9)		
Chemotherapy^d^				0.994	0.319			0.065	0.799			0.152	0.697
Yes	22	5 (22.7)	17 (77.3)			12 (54.5)	10 (45.5)			6 (27.3)	16 (72.7)		
No	31	11 (35.5)	20 (64.5)			18 (58.1)	13 (41.9)			10 (32.3)	21 (67.7)		
Surgery type				1.069	0.301			4.416	0.036			1.569	0.210
Wide resection	36	10 (27.8)	26 (72.2)			16 (44.4)	20 (55.6)			12 (33.3)	24 (66.7)		
Marginal resection	22	9 (40.9)	13 (59.1)			16 (72.7)	6 (27.3)			4 (18.2)	18 (81.8)		
Recurrence				1.046	0.306			1.384	0.239			0.429	0.513
Yes	33	9 (27.3)	24 (72.7)			16 (48.5)	17 (51.5)			8 (24.2)	25 (75.8)		
No	25	10 (40.0)	15 (60.0)			16 (64.0)	9 (36.0)			8 (32.0)	17 (68.0)		
Metastasis				0.834	0.361			1.277	0.258			0.840	0.359
Yes	20	5 (25.0)	15 (75.0)			9 (45.00)	11 (55.0)			7 (35.0)	13 (75.0)		
No	38	14 (36.8)	24 (63.2)			23 (60.5)	15 (39.5)			9 (23.7)	29 (76.3)		

*MPNST* malignant peripheral nerve sheath tumor, *CXCR4* C-X-C motif chemokine receptor 4, *CXCL12* C-X-C motif chemokine ligand 12, *AJCC* American Joint Committee on Cancer

^a^Tumor size information is missing for 1 patient

^b^AJCC stage information is missing for 3 patients

^c^Radiotherapy information is missing for 5 patients

^d^Chemotherapy information is missing for 5 patients

### CXCR4, CXCL12, and Cyclin D1 protein expression and their correlations

**Fig. 1:**
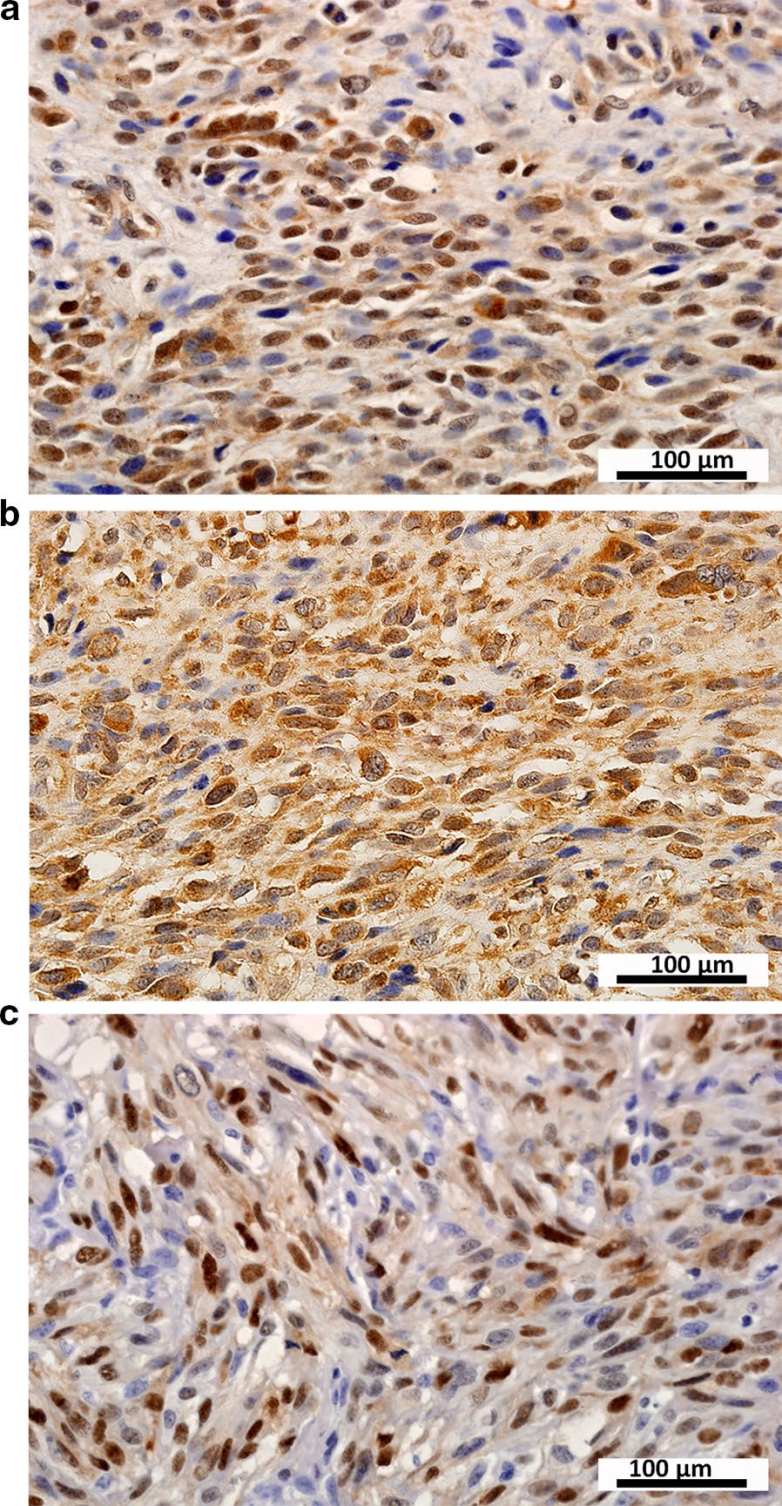
Immunohistochemical analysis of C-X-C motif chemokine receptor 4 (CXCR4), C-X-C motif chemokine ligand 12 (CXCL12), and Cyclin D1 protein expression in sporadic malignant peripheral nerve sheath tumor tissue samples. **a** CXCR4 is mainly expressed in the cytoplasm and membrane. **b** CXCL12 is mainly presented in the cytoplasm and plasma membrane. **c** Cyclin D1 is expressed in the nucleus

IHC analysis of MPNST samples revealed that CXCR4 was mainly expressed in the cytoplasm and membrane (Fig. 1a), CXCL12 was present in the cytoplasm and plasma membrane (Fig. 1b), and Cyclin D1 was predominantly localized in the nucleus (Fig. 1c). CXCR4, CXCL12, and Cyclin D1 were expressed at high levels in 19 (32.8%), 32 (55.2%), and 16 (27.6%) samples, respectively. Correlation analysis revealed positive correlations between CXCR4 expression and that of CXCL12 (*r* = 0.334, *P* = 0.010) and Cyclin D1 (*r* = 0.309, *P* = 0.018). However, there was no correlation between CXCL12 and Cyclin D1 expression (*r* = 0.091, *P* = 0.497).

### Relationships between CXCR4, CXCL12, and Cyclin D1 protein expression and clinicopathologic variables

CXCL12 expression was significantly associated with surgery type (χ^2^ = 4.416, *P* = 0.036); no significant associations were observed between clinicopathologic variables and CXCR4 or Cyclin D1 expression levels (Table 1).

### Relationships between CXCR4, CXCL12, and Cyclin D1 protein expression and survival

**Fig. 2:**
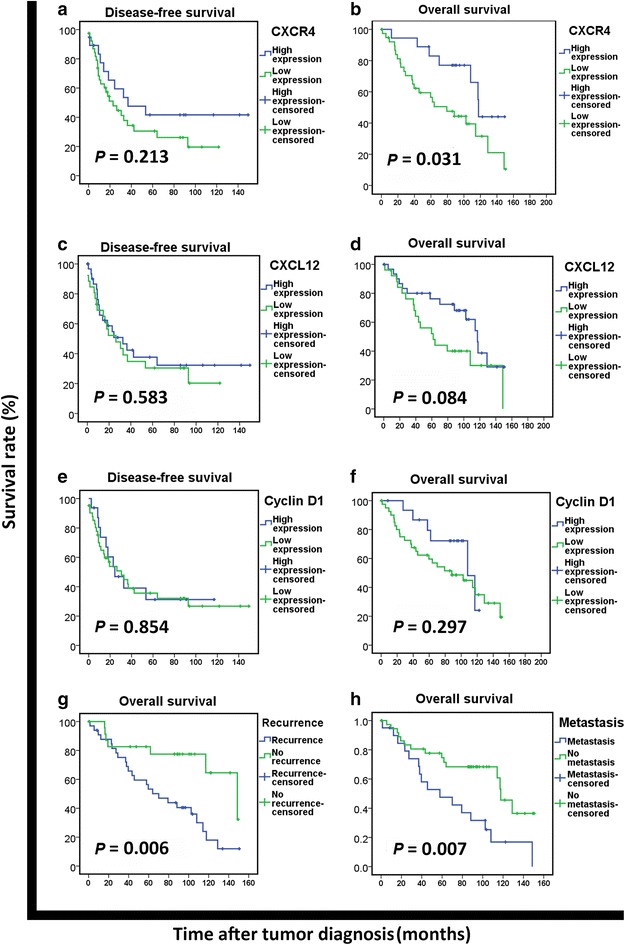
Prognostic values of CXCR4, CXCL12, Cyclin D1 expression, tumor recurrence, and tumor metastasis in sporadic malignant peripheral nerve sheath tumor patients. **a** No association is shown between CXCR4 expression and disease-free survival (DFS) (*P* > 0.05). **b** Overall survival (OS) is longer for patients with high CXCR4 expression than for those with low CXCR4 expression (*P* < 0.05). **c** No association is shown between CXCL12 expression and DFS (*P* > 0.05). **d** No association is shown between CXCL12 expression and OS (*P* > 0.05). **e** No association is shown between Cyclin D1 expression and DFS (*P* > 0.05). **f** No association is shown between Cyclin D1 expression and OS (*P* > 0.05). **g** OS is shorter for patients with recurrence than for those without recurrence (*P* < 0.01). **h** OS is shorter for patients with disease metastasis than for those without metastasis (*P* < 0.01)

Kaplan–Meier survival analysis indicated that high CXCR4 expression was not significantly associated with DFS (χ^2^ = 1.550, *P* = 0.213; Fig. 2a), but was associated with long OS (χ^2^ = 4.642, *P* = 0.031; Fig. 2b). High expression of CXCL12 and Cyclin D1 were not significantly associated with either DFS or OS (all *P* > 0.05, Fig. 2c–f). Patients who experienced recurrence or metastasis had significantly shorter OS than those who did not (both *P* < 0.01, Fig. 2g, h).

Univariate analysis indicated that tumor site, AJCC stage, recurrence, and metastasis were significant prognostic factors for DFS (all *P* < 0.05), whereas CXCR4 expression, recurrence, and metastasis were significant prognostic factors for OS (all *P* < 0.05; Table 2). However, neither CXCL12 nor Cyclin D1 had prognostic value as determined with univariate analysis (Table 2). Multivariate analysis revealed that recurrence and metastasis, but not CXCR4 expression, were independent prognostic factors for patients with sporadic MPNST (both *P* < 0.05; Table 3).

**Table 2:** Univariate analysis of the prognostic values of clinicopathologic characteristics and protein expression in sporadic MPNST patients

Parameter	Disease-free survival			Overall survival		
	HR	95% CI	*P*	HR	95% CI	*P*
Gender	0.791	0.409–1.530	0.486	1.203	0.585–2.472	0.616
Age group	1.003	0.508–1.983	0.992	0.628	0.280–1.407	0.258
Tumor site	1.606	1.004–2.571	*0.048*	0.882	0.540–1.440	0.615
Tumor size	1.199	0.792–1.814	0.391	0.972	0.610–1.548	0.905
AJCC stage	1.465	1.028–2.086	*0.034*	1.265	0.857–1.867	0.236
Radiotherapy	0.526	0.264–1.044	0.066	0.754	0.356–1.598	0.461
Chemotherapy	0.778	0.386–1.568	0.483	0.627	0.297–1.324	0.221
Surgical type	1.323	0.672–2.604	0.419	1.347	0.624–2.909	0.449
Recurrence	0.051	0.015–0.174	*< 0.001*	0.324	0.139–0.755	*0.009*
Metastasis	0.295	0.150–0.580	*< 0.001*	0.388	0.191–0.789	*0.009*
CXCR4	1.586	0.762–3.303	0.218	2.472	1.057–5.782	*0.037*
CXCL12	1.200	0.624–2.308	0.584	1.854	0.910–3.779	0.089
Cyclin D1	1.071	0.515–2.226	0.854	1.610	0.652–3.972	0.302

Italic values indicate statistically significant difference

*MPNST* malignant peripheral nerve sheath tumor, *AJCC* American Joint Committee on Cancer, *CXCR4* C-X-C motif chemokine receptor 4, *CXCL12* C-X-C motif chemokine ligand 12, *HR* hazard ratio, *CI* confidence interval

**Table 3:** Multivariate analysis of the prognostic values of clinicopathologic characteristics and protein expression in sporadic MPNST patients

Parameters	Disease-free survival			Overall survival		
	HR	95% CI	*P*	HR	95% CI	*P*
Tumor site	1.145	0.673–1.951	0.617	–	–	–
AJCC stage	0.942	0.597–1.488	0.799	–	–	–
Recurrence	0.062	0.018–0.215	*< 0.001*	0.378	0.159–0.899	*0.028*
Metastasis	0.558	0.222–1.400	0.214	0.447	0.215–0.928	*0.031*
CXCR4	–	–	–	1.961	0.829–4.639	0.125

Italic values indicate statistically significant difference

*MPNST* malignant peripheral nerve sheath tumor, *AJCC* American Joint Committee on Cancer, *CXCR4* C-X-C motif chemokine receptor 4, *HR* hazard ratio, *CI* confidence interval

## Discussion

In the present study, we identified a significant increase in CXCL4, CXCL12, and Cyclin D1 expression in tumor samples from Chinese patients with sporadic MPNST. CXCR4 expression was positively correlated with that of CXCL12 and Cyclin D1. Most importantly, patients with high CXCR4 expression had longer OS than did patients with low expression. High CXCR4 expression might define a specific sporadic MPNST subtype.

Our present data about the prognostic value of CXCR4 in sporadic MPNSTs was quite different from the reported data from NF1-associated MPNSTs [16], which showed that high CXCR4 expression was associated with poor prognosis. In NF1-associated MPNSTs, it was reported that CXCR4/CXCL12 signaling stimulated Cyclin D1 expression and enhanced MPNST cell proliferation which indicated poor prognosis [16]. Suppression of CXCR4 inhibited the growth of MPNST cell lines and tumorigenesis in allografts and spontaneous genetic mouse models of NF1-associated MPNST [16]. There are at least two possible explanations for the different prognostic values of CXCR4 in different subtypes of MPNST. One is that the pathogenesis of sporadic MPNST and NF1-associated MPNST may differ as a result of the quite different frequency of *NF1* mutations. While neurofibromin inhibits tumor cell proliferation through blockade of RAS-mediated signal transduction, it should also be considered a modulator of cell motility and cell adhesion [6]. Through interfacing with the cytoskeleton and membrane structures, neurofibromin acts as a negative regulator of the Rhodopsin/Rhodopsin-associated coiled-coil containing protein kinase (RHO/ROCK) signaling pathway involved in cytoskeletal dynamics that are instrumental in proper neuronal development [6]. The mutations of *NF1* cause neurofibromin to lose its functions, leading to poor prognosis of NF1-associated MPNSTs. However, sporadic MPNSTs show no or less frequent *NF1* mutations. Different pathogenesis might affect the role of CRCR4 in different subtypes of MPNSTs. A second possibility is that high CXCR4 expression might define a specific sporadic MPNST subtype. In this MPNST subtype, the typical phenotype is high CXCR4 expression which is associated with good prognosis. However, whether this phenotype represents a cause or a result is still unknown. Clearly, a more complete understanding of the role of CXCR4 in sporadic MPNSTs must await further study.

In the present study, we found that CXCR4 expression was positively correlated with that of CXCL12 and Cyclin D1, suggesting that CXCL12 and CXCR4 may act synergistically and interdependently to activate the downstream factor, Cyclin D1. However, although we found that CXCR4 expression was associated with OS of our patients, Cyclin D1 expression showed no apparent prognostic value. As a regulatory subunit of cyclin-dependent kinase 4 (CDK4) or cyclin-dependent kinase 6 (CDK6), Cyclin D1 activity is required for cell cycle G_1_/S transition, and many factors, including CXCR4, would regulate the expression and function of Cyclin D1. It is unclear whether CXCL12/CXCR4 signaling regulates Cyclin D1 in sporadic MPNSTs, as is the case in NF1-associated MPNSTs. We will use the human sporadic MPNST cell lines ST88-14 and T265p21 to investigate the role of CXCL12/CXCR4/Cyclin D1 signaling in this disease in our future study.

There are several limitations in our study. First, we did not examine the function of the CXCR4/CXCL12 signaling pathway, which is likely to shed light on the prognostic role of CXCR4 in sporadic MPNSTs. A functional study using sporadic MPNST cell lines might supply more data and clues. Second, we did not investigate the expression pattern or clinical significance of the CXCR4/CXCL12 signaling pathway in NF1-associated or radiotherapy-induced MPNST. However, these are rare disease subtypes, and we hope to perform such an analysis in the future when more samples are collected. Third, we did not investigate the influence of genetic/epigenetic factors on CXCL12 and CXCR4 expression.

In conclusion, CXCR4 expression is positively correlated with that of CXCL12 and Cyclin D1 and associated with OS in Chinese sporadic MPNST patients. High CXCR4 expression may define a specific sporadic MPNST subtype which is associated with better prognosis as compared with NF1-assciated MPNSTs.

## Abbreviations

AJCC: American Joint Committee on Cancer
CXCL12: chemokine (C-X-C motif) ligand 12
CXCR4: chemokine (C-X-C motif) receptor 4
DFS: disease-free survival
IHC: immunohistochemistry
MPNST: malignant peripheral nerve sheath tumor
NF1: neurofibromatosis type 1
OS: overall survival
TMA: tissue microarray
